# Green synthesis of gamma rays-induced melanin-based bismuth oxide nanoparticles for evaluation of the antibacterial and anti-virulence activities against extra-intestinal pathogenic bacteria

**DOI:** 10.1007/s11274-025-04533-1

**Published:** 2025-08-26

**Authors:** Amira Y. Mahfouz, Nermine N. Abed, Amira S. Abd-EL-Aziz, Rasha Mohammad Fathy

**Affiliations:** 1 Botany and Microbiology Department, Faculty of Science, Al-Azhar University (Girls Branch), Cairo, Egypt; 2 Drug Radiation Research Department, National Center for Radiation Research and Technology (NCRRT), Egyptian Atomic Energy Authority, Cairo, Egypt

**Keywords:** Gamma radiation, Bi_2_O_3_ NPs, *Citrobacter freundii*, *Enterobacter aerogenes*, Antibacterial, Antibiofilm

## Abstract

The rise of microorganisms that are resistant to drugs is one of the significant challenges facing the health sector. The need to identify effective alternatives facing bacterial diseases is significantly highlighted. Nanomaterials have the potential to be a game-changing weapon in the battle against infectious diseases. Melanin, with the assistance of different doses of gamma rays, successfully produced highly stable Bi_2_O_3_ NPs. The most effective dose was 40 kGy. Bi_2_O_3_ NPs were characterized using FTIR, XRD, DLS/zeta potential, and TEM. In this study, the antibacterial efficacy of Bi_2_O_3_ NPs versus the extra-intestinal bacteria *Citrobacter freundii* and *Enterobacter aerogenes* was evaluated. Bi_2_O_3_ NPs at 200 µL generate inhibition zone diameters of 35.26 ± 0.305 and 38.42 ± 0.570 mm for *C. freundii* and *E. aerogenes*, respectively. Bi_2_O_3_ NPs at 200 µL caused significant biofilm reduction of 81.05 and 88.25% also, registered a significant decline in the colony count of (0.29 × 10^8^) for *C. freundii* and (0.15 × 10^8^) for *E. aerogenes*. The Bi_2_O_3_ NPs greatly decreased catalase activity, displaying 1.5 ± 0.62 and 6.0 ± 0.91 U/mL, and lipase activity exhibiting 12.13 ± 0.34 and 6.7 ± 0.26 U/mL for *C. freundii* and *E. aerogenes*, respectively. SEM observations indicated that the bacterial cell membrane was disrupted, resulting in significant ultrastructure alterations. Bi_2_O_3_ NPs reported a moderate cytotoxic effect on normal kidney epithelial cells with IC_50_ value of 513.9 ± 2.71 µg/mL. In conclusion, Bi_2_O_3_ NPs exhibited potential antibacterial activity, suggesting recommended usage in medical fields. The novelty of this study is the use of low-dose gamma irradiation to synthesize highly stable Bi_2_O_3_ NPs.

## Introduction

Nanotechnology, an interdisciplinary field, offers various applications, including pharmacology, healthcare diagnostics, nutrition, chemistry, ecology, biological sciences, and energy production. Among the most frequently used metals in biomedicine are silver, gold, magnesium, copper, titanium, zinc, and bismuth (Bi) (Dizaj et al. 2015; Rudramurthy et al. 2016). Bismuth is a dipole magnetic semimetal with extremely narrow band gaps. Investigators have synthesized Bi NPs for electronic applications due to this material’s intriguing features, which include high magnetoresistance, thermal conductivity, and strong anisotropic electronic behavior (Wu et al. 2023a).

Bismuth (III) complexes have several medical applications. In this regard, bismuth subsalicylate treats diarrhea and upset stomach caused by overeating and drinking. Moreover, bismuth subcitrate potassium can be used in controlling *Helicobacter pylori* infection in combination with proton pump inhibitors and antibiotics. Bismuth nanoparticles have also been studied for their antibacterial, antifungal, antiparasitic, and antibiofilm properties (Gomez et al. 2021).

Although bismuth compounds are known to be effective against a variety of pathogens, little is known about their antimicrobial properties of bismuth nanoparticles (Vazquez-Munoz et al. 2020). Bi NPs offer a wide range of prospective applications in the biomedical field due to their low cost, lower toxicity, and remarkable properties (Gomez et al. 2021). Due to their amazing list of desirable characteristics, bismuth (Bi)-based nanoparticles (BBN) have generated a lot of attention as a major scientific breakthrough: high surface area, low chemical response, significant diamagnetism, excellent electrical and magneto resistance in a magnetic context, high stability, optimal catalytic activity, low cost, and ease of integration (Albadr et al. 2024).

Nanotechnology techniques can enhance bismuth stability and antibacterial activity because the structural and physicochemical characteristics of nanostructures are determined by controlling over size, structure, and environment. (Phan and Haes 2019) proposed that nanomaterials could be more stable than their bulk counterparts at a certain condition. Furthermore, capping compounds sustain the stability of nanoparticles, which is crucial for limiting their toxicity and prolonging their shelf lifespan. In the past 20 years, metallic substances with proven or probable antibacterial characteristics (e.g., silver, copper, and titanium) have been employed to create antimicrobial nanoparticles (Vazquez-Munoz and Lopez-Ribot 2020).

Bi-based medications are applied for treating a variety of gastrointestinal disorders, including gastric ulcer infections and dyspepsia. In addition to potential treatments for tumors, multidrug-resistant (MDR) diseases, and viral diseases, their therapeutic applications have recently expanded to include drug delivery, assays, and biosensors (Vazquez-Munoz and Lopez-Ribot 2020). Because Bi subsalicylate has been prescribed to treat stomach disorders and since there are no findings of any negative consequences from exposure to Bi NPs, they are thought to be safe for human cells. Recent studies have demonstrated that certain Bi-based compounds and Bi NPs exhibit an antibacterial effect versus *Pseudomonas aeruginosa*,* Staphylococcus aureus*,* H. pylori*, and *Escherichia coli.* Bi-compounds have been utilized for decades as antibacterial agents (Wu et al. 2023a).

Bi_2_O_3_ NPs showed significant antibacterial effects by disrupting biofilm formation, enzyme activity, and cell morphology, indicating their biological efficacy against resistant pathogens. Clinically, their broad-spectrum activity and potential low toxicity suggest applications in antimicrobial coatings, wound care, and adjunct therapies, particularly for hospital-acquired infections (Bartoli et al. 2020).

One of the biggest challenges facing public health globally is the rise of drug-resistant bacteria. Antibiotics are the traditional treatment and preventative measure for microbial infections, but they are no longer effective due to the rise of antimicrobial resistance (AMR) (Freeland et al. 2023). According to estimates, 4.73 million people will die each year from diseases brought on by AMR microbes by 2050, with a potential $100 trillion worldwide economic effect (O’Neill 2015).

Despite the widespread use of current antimicrobial therapies, several critical limitations persist. The foremost issue is the rise of antimicrobial resistance (AMR), where pathogens such as *Klebsiella pneumoniae*, and *P. aeruginosa* have developed resistance to multiple drug classes due to misuse and overuse of antibiotics (Organization 2024). Additionally, many antibiotics exhibit broad-spectrum activity, disrupting the host microbiota and causing dysbiosis and secondary infections (e.g., *Clostridium difficile*) (Francino 2016). Traditional antimicrobials also show limited efficacy against bacterial biofilms, which are significantly more resistant to treatment. Moreover, some agents can induce cytotoxic effects and allergic reactions, especially at higher concentrations (Wang et al. 2017). The discovery rate of new antibiotics remains slow, largely due to high development costs and low commercial incentives. Collectively, these challenges underscore the urgent need for alternative antimicrobial strategies such as nanomaterials and targeted therapies (Silver 2011).

Urinary tract infections (UTIs) are among the most common bacterial infections in hospitals and communities, and their morbidity and mortality rates have recently raised globally. In recent years, antimicrobial resistance has emerged as a general problem, particularly among urinary tract pathogens such as *Morganella morganii*,* Citrobacter freundii*,* P. aeruginosa*,* Escherichia coli*, and *Enterobacter aerogenes* (Mohapatra et al. 2022).

*Citrobacter* species belong to the Enterobacteriaceae family of facultative anaerobic Gram-negative bacteria. Urinary tract infections (UTIs), pneumonia, septicemia, or common bacteremia have all been linked to nosocomial infections caused by the opportunistic invasive bacterium *Citrobacter* (Jabeen et al. 2023). *Enterobacter* is a genus of Gram-negative, rod-shaped, non-spore-forming, facultative anaerobic bacteria belonging to the family *Enterobacteriaceae*. *Enterobacter aerogenes* has gained clinical significance as an opportunistic pathogen and has emerged as a nosocomial pathogen in intensive care patients (Mezzatesta et al. 2012).

This work aimed to elucidate the antibacterial activity of melanin-based Bi_2_O_3_ NPs against the extra-intestinal pathogenic bacteria *Citrobacter freundii* and *Enterobacter aerogenes*, and to study the effect of melanin-based Bi_2_O_3_ NPs in combating the disease-associated virulence factors of these clinically relevant bacterial strains.

## Materials and methods

### Chemicals and reagents

The *Curvularia* sp. AS21 ON076460 melanin pigment that was extracted and published in our previous study (Abd-EL-Aziz et al. 2024) was used in the biosynthesis of Bi_2_O_3_ NPs. The analytical pure chemicals utilized in this work were obtained from Difco (United Kingdom) and Sigma-Aldrich (St. Louis, Missouri, 6s3103, USA).

### Synthesis of Bi_2_O_3_ NPs

**Fig. 1:**
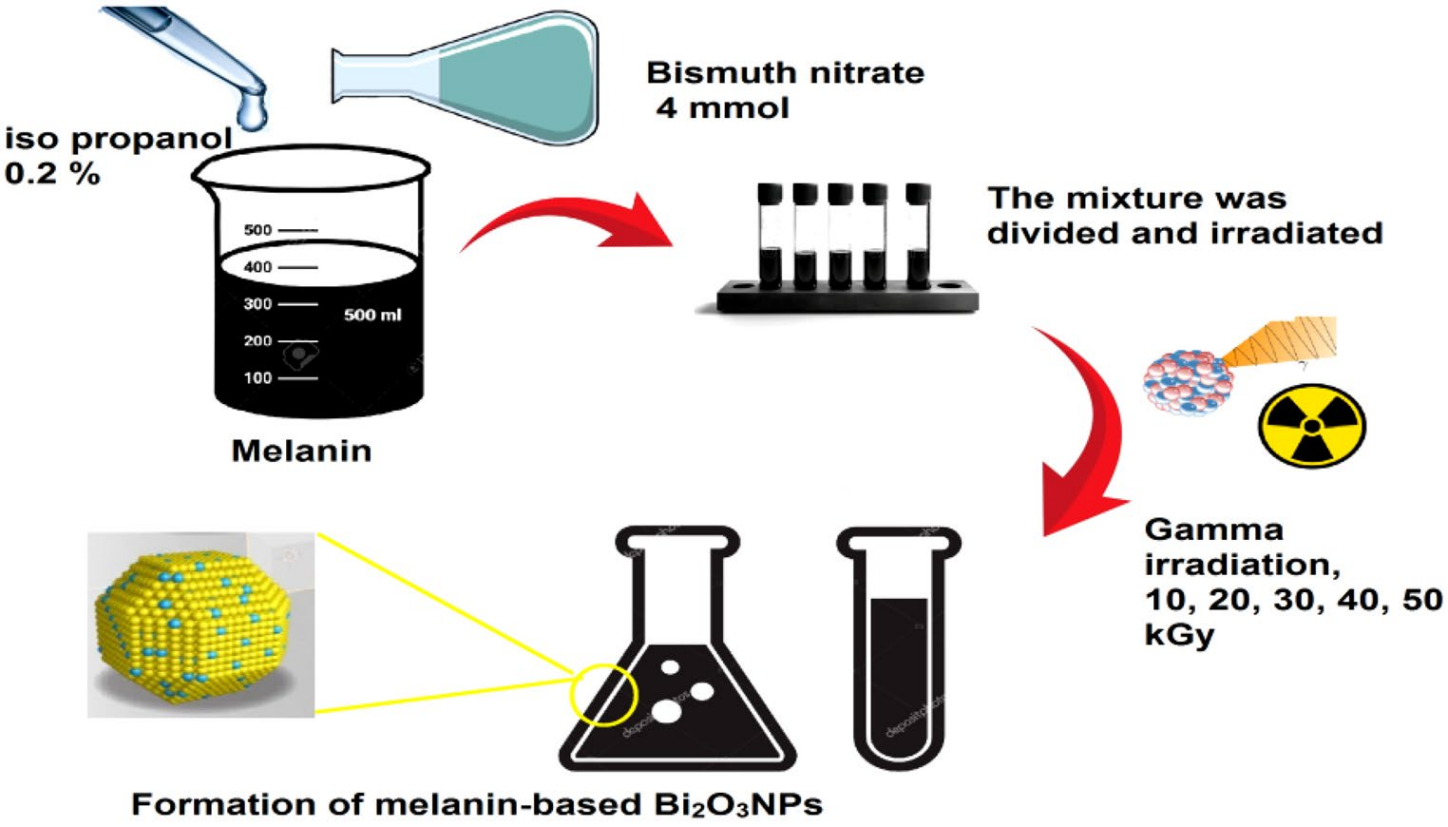
The schematic diagram of melanin-based Bi_2_O_3_ NPs synthesis

About (6.493 mg/mL) of melanin pigment was mixed with 4.0 mmoL of bismuth nitrate solution in a 1:5 (v/v) ratio with 0.2% isopropanol as a free radical scavenger. The mixtures were irradiated at different gamma doses, 10, 20, 30, 40, and 50 kGy. The irradiation was done at the National Center for Radiation Research and Technology (NCRRT) in Cairo, Egypt, using ^60^Co gamma-cell sources at a dosage rate of 0.986 kGyh^− 1^. A sample with high optical density (O.D.) was selected for further investigation. The schematic diagram of melanin-based Bi_2_O_3_ NPs synthesis was illustrated in Fig. 1.

### Characterization of melanin-based Bi_2_O_3_ NPs

The UV-Visible spectrophotometer (T60 UV-Vis-UK) was used to characterize melanin-based Bi_2_O_3_ NPs. The Fourier-transform infrared spectroscopy (FT-IR) analysis was performed using a Bruker-Germany FTIR-Vertex 70 instrument which employed pellets of potassium bromide (KBr) compacted with dried samples. Dynamic light scattering (DLS-PSS-NICOMP 80-ZLS particle sizing system, St. Barbara, CA, USA) was utilized to evaluate the particle distribution and size. The X-ray diffraction analysis was examined using the (XRD 6000 series), counting residual crystallite size/lattice and crystal nature (Shimadzu apparatus using Cu-K (α), λ꞊ 1.5418 Å and nickel filter, Shimadzu Scientific Instruments (SSI), Tokyo, Japan). The biosynthesized Bi_2_O_3_ NPs size and morphology were examined using a high-resolution transmission electron microscope (TSEM, Thermo Fisher, Holand).

### Source of the pathogenic bacteria

Clinical isolates *Citrobacter* sp. and *Enterobacter* sp. were kindly obtained from the Microbiology Laboratory at Zefta General Hospital, Gharbia Governorate, Egypt. The isolates were identified at the species level by Vitek 2 compact automated microbial identification (bioM´erieux, Marcy l’Etoile, France) using GN cards test panels (ID-GPC) (bioM´erieux) (Salama 2024). The bacterial cultures were maintained on nutrient agar slants and sub-cultured every 14 days.

### The antibiotic susceptibility test

*Citrobacter* sp. and *Enterobacter* sp. were evaluated for sensitivity or resistance to 11 antibiotics categorized into six classes using the disk diffusion procedure as described by (Mohammed et al. 2024). The tested antibiotics were: Streptomycin (S, 10 µg), Amikacin (Ak, 30 µg), Ceftriaxone (CTR, 30 µg), Vancomycin (VA, 30 µg), Ofloxacin (OF, 5 µg), Amoxicillin (AX, 25 µg), Colistin (CL, 10 µg), Novobiocin (NV, 5 µg), Doxycycline (DO, 30 µg), Gentamicin (GEN, 10 µg), and Ampicillin (AMP, 19 µg). Briefly, the bacterial isolates *Citrobacter* sp. and *Enterobacter* sp. were inoculated on a Nutrient agar medium (Oxoid Ltd, England). The antibiotic discs were placed on the surfaces of the inoculated plates and incubated for 24 h at 37 °C. The zones of inhibition (ZOIs) surrounding the antibiotic discs were measured in millimeters to assess bacterial growth rates.

### The assessment of the antibacterial activity of melanin-based Bi_2_O_3_ NPs

The antibacterial efficiency of melanin-based Bi_2_O_3_ NPs was verified by the agar well diffusion assay against *Citrobacter* sp. and *Enterobacter* sp. The bacterial inoculum (1.5 × 10^5^ CFU/mL) was inoculated on the surfaces of agar plates. Over the agar surface, wells of 6.0 mm in width were formed. A 200 µL of melanin-based Bi_2_O_3_ NPs was added into every well and permitted to stand for an hour for successful diffusion. Ampicillin (40 µg) was applied as a positive control. Following 24 h of incubation at 37º C, the (ZOIs) were detected (Mousaa et al. 2022).

### Determination of minimum inhibitory concentration (MIC) of melanin-based Bi_2_O_3_ NPs

The MIC assessment was done following the methods of (Salem et al. 2021). The bacterial inoculum (1.5 × 10^4^ CFU/mL) was added to the nutrient agar plates’ surface that were first implemented with melanin-based Bi_2_O_3_ NPs at concentrations of 3.125 up to 100 µL and then incubated at 37 ˚C for 24 h. The experiment was performed in triplicate. The MICs were defined as the lowest concentrations at which no bacterial growth was visible on the surface of agar plates.

### Determination of minimum bactericidal concentration (MBC) of melanin-based Bi_2_O_3_ NPs

The MBC of melanin-based Bi_2_O_3_ NPs was estimated. Briefly, 0.5 McFarland’s standard (1.5 × 10^8^ CFU/mL) of the tested bacterial isolates was inoculated on the nutrient broth medium with the concentrations of the melanin-based Bi_2_O_3_ NPs that did not exhibit any discernible growth in the MIC assessment (those at or above the MIC) and then incubated for 24 h at 37 ° C. Next, 10 µL of the broth dilutions were streaked onto the agar and incubated for 24 to 48 h. The specimen that did not exhibit any discernible growth was detected as the MBC.

### The antibiofilm activity of melanin-based Bi_2_O_3_ NPs

The antibiofilm efficacy of melanin-based Bi_2_O_3_ NPs against the tested bacterial strains was assessed using the control-by-tubes assay method as described by (Fathy et al. 2020b). Ten microliters of 0.5 McFarland (1.5 × 10^8^ CFU/mL) of the bacteria under investigation were introduced into test tubes along with 5 mL of nutrient broth. Next, different concentrations (25, 50, 100, and 200 µL) of melanin-based Bi_2_O_3_ NPs were included separately in the inoculated nutrient broth and incubated for 24 h at 37 °C. The media components of the control and treated tubes were discarded, followed by treatment with phosphate buffer saline (PBS) pH 7. The adhesive bacterial layers were then stabilized for 10 min with 5.0 mL of sodium acetate (3.0%), followed by a wash with deionized water. After 15 min of staining with crystal violet (0.1%), biofilms were washed out with deionized water to remove any remaining stain. Lastly, the crystal violet stain attached to the bacterial colonies was dissolved with ethanol. The T60 U-UV-Vis-UK UV-Visible spectrophotometer was employed to quantitatively assess the inhibition of bacterial biofilm formation at λ = 570 nm. The subsequent equation (Eq. 1.) was executed to determine the inhibition percent according to (El-Nemr et al. 2020; Sultan et al. 2024).

1 $$\:\text{I}\text{n}\text{h}\text{i}\text{b}\text{i}\text{t}\text{i}\text{o}\text{n}\:\%=\:\frac{\text{O}\text{D}\text{c}\:-\:\text{O}\text{D}\text{t}}{\text{O}\text{D}\text{c}}\times\:100$$

Where, ODc: The absorbance of the control sample, ODt: The absorbance of the treated samples.

### The impact of melanin-based Bi_2_O_3_ NPs on the viable colony counts

The viable colony count assay of bacteria treated with melanin-based Bi_2_O_3_NPs was carried out (Mohammed et al. 2024). The tested bacteria at the exponential growth phase were grown in nutrient broth (NB) to obtain 1.5 × 10^6^ CFU/mL concentration of the bacterial suspension. The 4 × MIC of melanin-based Bi_2_O_3_ NPs was applied. Samples were serially diluted from 10^− 1^ to 10^− 6^ before being inoculated on nutrient agar medium for colony enumeration. All experiments were done in triplicate. The CFU/mL and log_10_ of bacteria were calculated.

### Determination of melanin-based Bi_2_O_3_ NPs efficiency on catalase activity (CAT)

To analyze the rate at which hydrogen peroxide (H_2_O_2_) was consumed, the catalase activity was evaluated in the melanin-based Bi_2_O_3_NPs- treated and non-treated bacteria. Catalase activity was measured spectrophotometrically at λ = 240 nm by monitoring the decline in H_2_O_2_ concentration (Lee et al. 2018). A cuvette containing 2.89 mL of 50 mM potassium phosphate buffer (pH-7.4) was loaded with 10 µL of the cell-free lysate at time zero. Next, 0.1 mL of 300 mM H_2_O_2_ was incorporated. For three minutes, the absorbance at 240 nm was measured at 1.0-minute intervals. Catalase activity was calculated from the subsequent equation (Eq. 2):

2 $$\eqalign{& {\rm{Catalase activity}}\left( {{{{\rm{Unit}}} \over {{\rm{mL}}}}} \right) \cr& = {{{\rm{\Delta Abs}} \times {\rm{Total volume assay}}} \over {{\rm{\Delta t}} \times {\rm{\varepsilon }} \times {\rm{l}} \times {\rm{Enzyme sample volume}}}} \cr} $$

Where, ΔAbs is the change in absorbance, Δt is the time of incubation (min), ε is the extinction coefficient of substrate in units of M-1 cm^− 1^, and l is the cuvette diameter (1 cm).

### Determination of melanin-based Bi_2_O_3_ NPs efficiency on lipase activity

The activity of lipase was evaluated spectrophotometrically using p-nitrophenylpalmitate (p-NPP) as a substrate (Amin et al. 2022). To prepare the substrate solution, 16 mM of p-NPP in 2-propanol was mixed with 4.5 mL of 50 mM Tris-HCl buffer (pH 8.0). 100 µL of the bacterial supernatants (control and treated melanin-based Bi_2_O_3_NPs at concentrations of 0, 25, 50, 100, and 200 µL) were mixed with 400 µL of p-NPP. The reaction was stopped by the addition of 30 µL of absolute ethanol. The absorbance of the mixtures was estimated at λ꞊ 410 nm. One unit of the enzyme activity was defined as the quantity of enzyme required releasing one mmoL of p-nitrophenyl per 1 min under test conditions. The activity (U/mL) was calculated according to the equation (Eq. 3):

3 $$\:\text{L}\text{i}\text{p}\text{a}\text{s}\text{e}\:\text{a}\text{c}\text{t}\text{i}\text{v}\text{i}\text{t}\text{y}\:\left(\text{U}/\text{m}\text{L}\right)=\:\frac{{\left({\text{A}}_{\text{t}}{-\text{A}}_{\text{b}}\right)\times\:}_{}{\text{V}}_{\text{t}\text{o}\text{t}\text{a}\text{l}\:}\times\:\text{d}\text{f}}{\left(0.0148\right)\times\:{\text{V}}_{\text{e}\text{n}\text{z}}}$$

Where, ∆A_t_is the absorbance of the sample. ∆A_b_is the absorbance of the blank. V_total_ is the total volume of the assay. df is the dilution factor. (0.0148) is the extinction coefficient of p-NPP at 410 nm. V_enz_is the volume of enzyme used.

### Electron microscopic analysis

The electron microscopic analysis of melanin-based Bi_2_O_3_ NPs treated and untreated bacteria were examined. The treated bacterial suspension (10^7^ CFU/mL) was treated with 4×MIC of Bi_2_O_3_ NPs for 18 h. The control bacterial samples were applied without Bi_2_O_3_ NPs.

### Cytotoxicity of the melanin-based Bi_2_O_3_ NPs on the Vero cells using MTT protocol

Vero cells (ATCC CCL-81), epithelial, adherent, and non-tumorigenic cells derived from the kidney of the African green monkey *Cercopithecus aethiops*, were used for the cytotoxicity assay. To assess the cytotoxic potential of the melanin-based Bi_2_O_3_ NPs, a 96-well tissue culture plate was seeded with 1 × 10^6^ cells/mL (100 ML per well) and incubated at 37 °C for 24 h to establish a uniform monolayer. Once confluence was achieved, the culture medium was aspirated, and the wells were gently rinsed twice with a washing buffer. Serial two-fold dilutions of the melanin-based Bi_2_O_3_ NPs were prepared in RPMI medium supplemented with 2% serum and dispensed into designated wells, while control wells received only the maintenance medium. The plate was then incubated at 37 °C. Next, 20 µL of MTT solution (5 mg/mL in PBS) was added to each well, and the plate was placed on a shaker at 150 rpm for 5 min to ensure thorough dispersion. The cells were further incubated at 37 °C in a 5% CO₂ for 4 h to facilitate MTT reduction. Thereafter, the medium was removed, and the insoluble formazan crystals were dissolved in 200 µL of DMSO. Absorbance was recorded at λ = 560 nm, where the measured optical density reflected the number of viable cells (Algotiml et al. 2022).

### Statistical analysis

The results of all tests of the biological efficiency were regarded as (Mean ± SD). The statistical examines were accomplished by one way ANOVA at *P* < 0.05. The antimicrobial results were recorded as least significant difference (LSD) and Duncan’s multiple range analyses employing the SPSS (IBM) software (V24).

## Results and discussion

### Characterization of melanin Bi_2_O_3_ NPs

#### UV–Visible spectrophotometer analysis

**Fig. 2:**
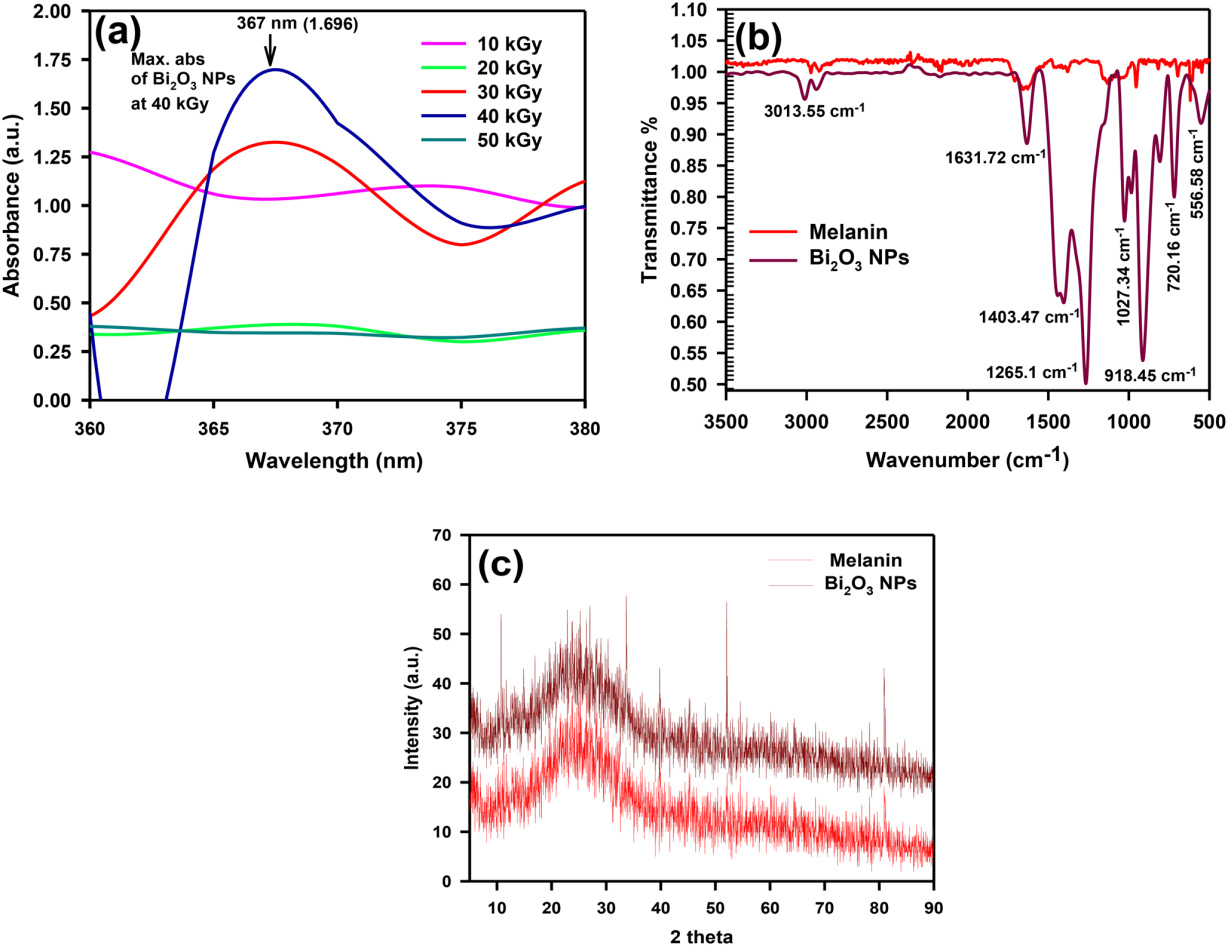
Characterization of melanin-based Bi_2_O_3_ NPs (**a**) UV–Vis spectrophotometer, (**b**) FTIR and, (**c**) XRD patterns

Fabrication of green synthesized nanomaterials is becoming increasingly common and has been suggested as a substitute for both physical and chemical methods (Hosny et al. 2024). As demonstrated in Fig. 2a, the UV-visible spectrum of melanin-based Bi_2_O_3_ NPs synthesized using gamma irradiation and the proximity of melanin pigment indicate that the most effective dose in Bi_2_O_3_ NPs production is 40 kGy. The results indicate a maximum absorbance of 1.696 at λ = 367 nm, which confirms the formation of melanin-based Bi_2_O_3_ NPs (Kashif et al. 2023; Mohammadi et al. 2019; Shaker et al. 2022).

The FTIR technique is an effective analysis to quantitatively examine interactions between the bonds in certain compounds. The FT-IR spectra of melanin-based Bi_2_O_3_ NPs (Fig. 2b) exhibit a distinctive absorbance band at melanin peaks were identified at 3100 cm^− 1^, 2922 cm^− 1^, 1625 cm^− 1^, 1400 cm^− 1^ to 1500 cm^− 1^, and 1015 cm^− 1^, which were consistent with the findings of the previous study (Abd-EL-Aziz et al. 2024). Furthermore, the band at 2922 cm^− 1^ corresponds to stretching vibrations of aliphatic C-H bonds. The vibrations of aromatic ring C = O groups are responsible for the band at 1625 cm^− 1^, whereas aliphatic C = C groups may cause bands between 1400 and 1500 cm^− 1^. The band at 1015 cm^− 1^ corresponds to the C-O group. In the melanin characterization section, the interpretation of the remaining FTIR peaks was explored. Melanin-based Bi_2_O_3_ NPs show modest variations in transmittance%, with a new peak at 556.58 cm^− 1^ due to BiO symmetrical stretching vibration (Sánchez-Martínez et al. 2016). By correlating the FT-IR of Bi_2_O_3_ NPs incorporated with melanin, changes observed within the bands of the extracted melanin could be linked to an alteration in the force constant (k) of the compounds due to adsorption of the melanin carboxylate group on the bismuth oxide particles. The difference in peaks was attributed to the carboxylate group, Ce O stretch vibration, and primary amine (NeH), proving that the produced melanin effectively reduces bismuth nitrate and melanin-stabilized Bi_2_O_3_ NPs.

#### X-ray diffraction (XRD)

XRD offers an accurate indication of the structure and composition of the analyzed particles by revealing the axis, size, and atom status (El-Batal et al. 2017). Figure 2c shows the XRD design of melanin-based Bi_2_O_3_ NPs; several peaks were observed, including those for melanin-based Bi_2_O_3_ NPs. Diffraction properties are seen through 2 theta (degree) as 10.74°, 26.99°, 29.05°, 33.67°, 39.80°, 45.2°,47.3°, 52.04°, 54.3° and 80.90° (Badireddy et al. 2014) corresponding to the crystalline planes of (201), (101), (002), (220), (321), (222), (400), (402), (203), and (113), respectively which closely corresponds to the typical JCPDS card (JCPDS NO. 27–0050). The existence of those peaks correlates to nanoparticles; consequently, the XRD analysis indicates that the melanin-based Bi_2_O_3_NPs are extensively crystallized and form a face-centered cubic (fcc) structure. The average crystalline size of the Bi_2_O_3_ NPs estimated by the Debye–Scherrer equation D = kλ/βcosθ was found to be 19.55 nm. The attained XRD peak validates the tetragonal phase of the synthesized Bi_2_O_3_ NPs.

#### DLS and zeta potential

**Fig. 3:**
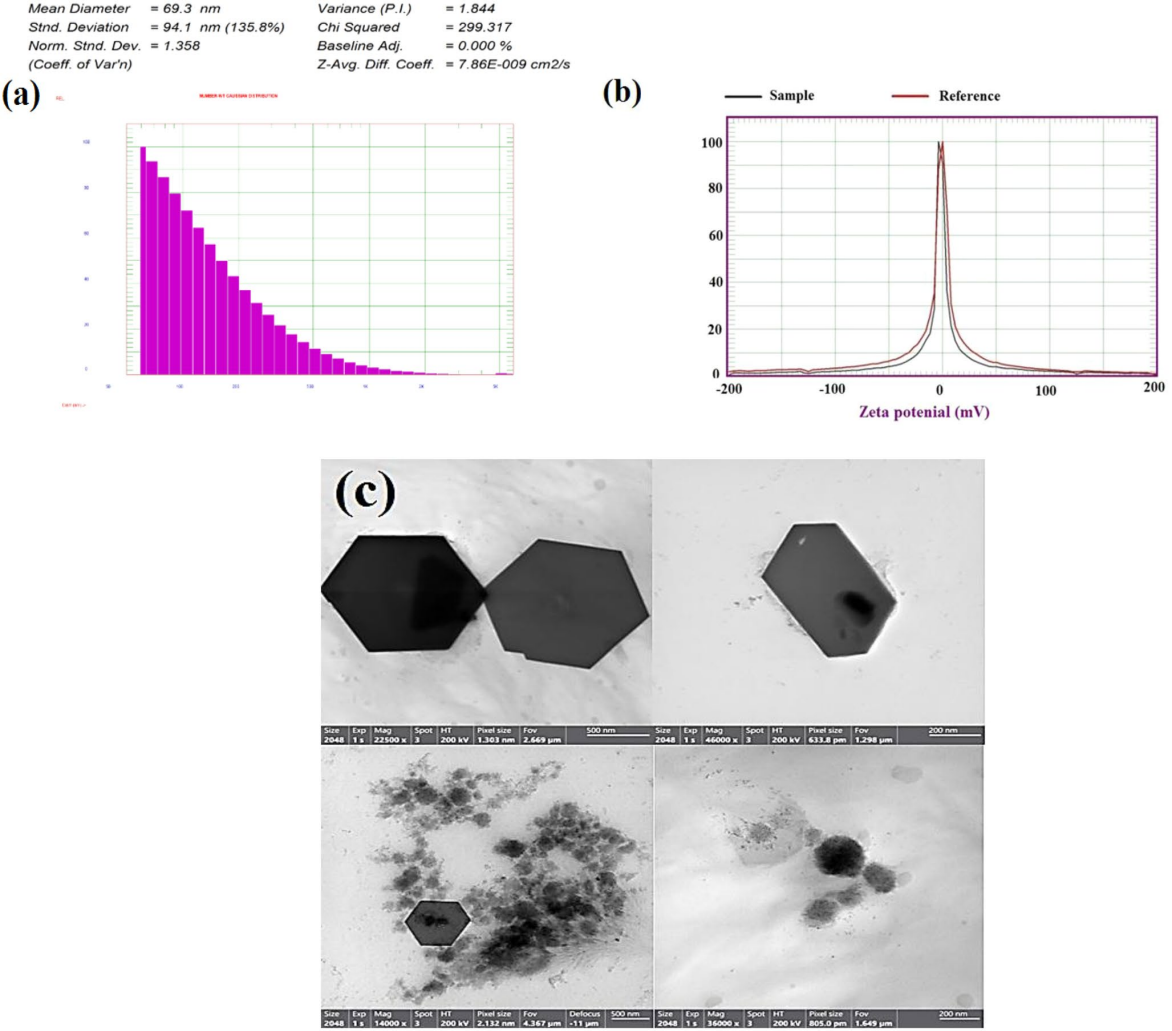
Characterization of melanin-based Bi_2_O_3_ NPs (**a**) DLS, (**b**) Zeta potential, and (**c**) TEM images

The estimation of particle size dissemination and the average particle size was determined using DLS analysis. The size distribution of melanin-based Bi_2_O_3_ NPs using the DLS is shown in Fig. 3a. The observed mean sizes of melanin-based Bi_2_O_3_ NPs ranging 69.3 ± 1.35 nm. It was necessary to clarify that the mono-distributed Bi_2_O_3_ NPs is due to the presence of melanin, which functioned as an excellent capping and stabilizing agent. Additionally, the synthesized Bi_2_O_3_NPs have non-uniform shapes and sizes, which verify polydispersed nanoparticles [85], as seen in DLS. Moreover, the Zeta potential was measured − 4.73 mV, respectively, which indicates the proper stability and low aggregation of the NPs (Fig. 3b).

TEM images, as represented in Fig. 3c, show that the biosynthesized Bi_2_O_3_NPs were doped on the surface of the melanin particles. The synthesized melanin-based Bi_2_O_3_NPs are spherical. The particles are within the nanoscale with an average diameter of 20.5 ± 1.7 nm. It has revealed that Bi_2_O_3_ NPs are loaded tightly on melanin.

#### Identification of the bacterial isolates

In the current study, the clinical isolates obtained from Zefta General Hospital were identified using the Vitek 2 compact automated microbial identification system as *C. freundii* and *E. aerogenes*. The study commenced with the assessment of the antibacterial activity of the melanin-based Bi_2_O_3_ NPs, followed by antibiotic susceptibility testing of these isolates to evaluate their resistance profiles.

#### The antibiotic susceptibility test

**Fig. 4:**
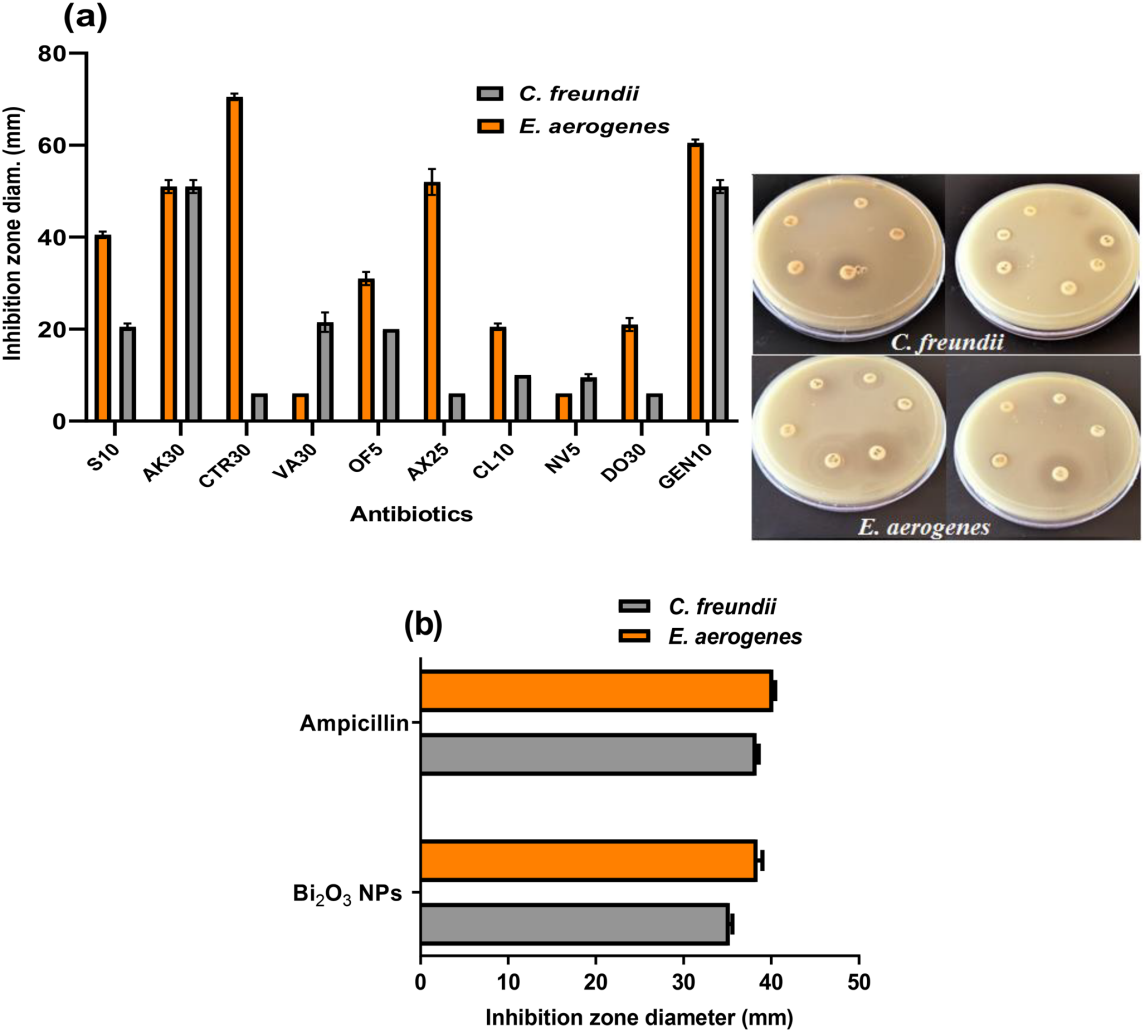
The antibacterial activity of melanin-based Bi_2_O_3_ NPs on *C. freundii* and *E. aerogenes* (**a**) The antibiotic susceptibility test, (**b**) Well diffusion assay at a concentration of 200 µL

The bacterial strains were defined as multi-drug-resistant (MDR) if they were not susceptible to one compound from three or more antimicrobial groups according to (Magiorakos et al. 2012). An antibiotic susceptibility test was performed on *C. freundii* and *E. aerogenes* using 11 different antibiotics. The findings reveal that the majority of antibiotics used have moderate or no effect on inhibiting the growth of *C. freundii* and *E. aerogenes*. Table 1 shows that, these antibiotics belong to separate classes and functions through different mechanisms of moderate or no action on *C. freundii* and *E. aerogenes.* It was found that *C. freundii* is more resistant to the tested antibiotics than *E. aerogenes*. As shown in Fig. 4a, the most potent antibiotics for *C. freundii* are Amikacin Ak30 and Gentamicin GEN10 record inhibition zone diameters of 50.26 ± 0.75, and 50.91 ± 1.23 mm, respectively. While with *E. aerogenes* the most influential antibiotics are Ceftriaxone CTR30, Gentamicin GEN10, Amikacin Ak30, and Amoxicillin AX25 record inhibition zone diameters of 70.0 ± 1.36, 61.25 ± 0.57, 50 ± 1.47, and 49.57 ± 0.34 mm, respectively.

Most urinary tract isolates were resistant to norfloxacin, cefotaxime, cephalexin, ciprofloxacin, and aminoglycosides (Metri et al. 2013). According to reports, 39–48% of *C. freundii* isolates are resistant to wide-spectrum cephalosporins (ceftriaxone, ceftazidime), piperacillin, and piperacillin/tazobactam (Khorasani et al. 2008). On the other hand, *Citrobacter* sp. is usually susceptible to third-generation cephalosporins, carbapenem, and colistin. *C. freundii* frequently exhibits resistance to first- and second-generation cephalosporins as well as β-lactam drugs (Arana et al. 2017; Maraki et al. 2017). There have been more reports of antibiotic-resistant *Citrobacter* isolates occurring globally (Osei Sekyere et al. 2021).

*Citrobacter* species have many antibiotic-resistant genes that are expressed in their chromosomes or plasmids (Huang et al. 2023). Similar to this, *Enterobacter* species exhibit inherent resistance to ampicillin and wide-spectrum cephalosporins. Additionally, they have acquired genetic mobile elements that have made them resistant to numerous antimicrobial agents, including carbapenems and third-generation cephalosporins, making it challenging to determine the most effective course of treatment (Intra et al. 2023). Furthermore, *Enterobacter* species have an inherent AmpC β-lactamaseso they are not susceptible to ampicillin, amoxicillin, first-generation cephalosporins, and cefoxitin (Davin-Regli et al. 2019; Quraishi et al. 2019).

**Table 1:** The sensitivity of *C. freundii and E. aerogenes* to different 11 antibiotics

Antibiotic	*C. freundii*	*E. aerogenes*
Streptomycin S10	intermediate	Sensitive
Amikacin Ak30	sensitive	Sensitive
Ceftriaxone CTR30	resistant	sensitive
Vancomycin VA30	intermediate	resistant
Ofloxacin OF5	intermediate	intermediate
Amoxicillin AX25	resistant	sensitive
Colistin CL10	intermediate	intermediate
NovobiocinNV5	intermediate	resistant
Doxycycline DO30	resistant	intermediate
Gentamicin GEN10	sensitive	sensitive
Ampicillin AMP19	resistant	resistant

### The assessment of the antibacterial activity of melanin-based Bi_2_O_3_ NPs against *C. freundii* and *E. aerogenes*

The antimicrobial efficiency of Bi_2_O_3_ NPs against *C. freundii* and *E. aerogenes* was assessed. As denoted in Fig. 4b, the incorporated melanin-based Bi_2_O_3_ NPs display a significant antimicrobial potential toward *C. freundii* and *E. aerogenes* at 200 µL with inhibition zone diameters (ZOIs) of about 35.26 ± 0.305 and 38.42 ± 0.570 mm, respectively, compared to 38.30 ± 0.26 and 40.24 ± 0.23 mm gained by standard antibacterial ampicillin.

Various metal and metal oxide nanoparticles, such as Ag, TiO_2_, ZnO, CuO, and MgO are well-established for their antibacterial activities as summarized in Table 2, although Bi_2_O_3_ NPs have recently emerged as promising alternatives due to their unique physicochemical properties. Bi_2_O_3_ NPs exhibit notable antimicrobial, anticancer, and photocatalytic activities while maintaining relatively lower toxicity profiles compared to silver or copper-based NPs (Li et al. 2022). Moreover, bismuth compounds have a long-standing history of use in medicine (e.g., in gastrointestinal treatments), which supports their biocompatibility (Ganapathy et al. 2022). The eco-friendly synthesis of Bi_2_O_3_ NPs using melanin and gamma irradiation also adds to their appeal as a green nanomaterial. Therefore, Bi_2_O_3_ NPs offer a safe, effective, and sustainable option in the development of new antibacterial agents (Ahmed et al. 2016).

**Table 2:** Comparative of metal oxide nanoparticles in bio therapy

Nanoparticles	Biosynthesizing	Bio therapy	Representative studies
ZnO	Using *Calotropis gigantea* leaf extract	Excellent bactericidal activity against skin ulcer pathogens, *Staphylococcus aureus.*	(Govindasamy et al. 2021)
TiO_2_	Using the endophytic fungus *Aspergillus* sp.	Have a potential anticancer effect	(Alhadrami et al. 2025)
Ag	Using the fungus, *Gliocladium deliquescens*	Ag NPs showed functional antimicrobial activity against *Salmonella* sp. and *Staphylococcus* sp.	(Fathy et al. 2020a)
CuO	Using *Calotropis gigantean* plant extract	Strong antibacterial against *S. aureus*,* Escherichia coli*, and MRSA.	(Govindasamy et al. 2023b)
TiO_2_/ZnO	Using *Urtica Smensis* leaf extract	High antibacterial potential against four bacterial strains of *S. aureus*,* Streptococcus pyogenes*,* Pseudomonas aeruginosa*, and *Escherichia coli.*	(Atiek et al. 2024)
CuO/TiO_2_	Encapsulation of CuO-TiO_2_ particles in the polypropylene matrix	Excellent antimicrobial efficacy towards *E. coli* and *S. aureus*, mortality infections from catheters.	(Govindasamy et al. 2024a, b)
ZnO/CuO	Using *Calotropis gigantea* leaf extract	Promising wound healing management.	(Govindasamy et al. 2022, 2023a)
Bi_2_O_3_	Using cell-free extract of *Spirulina platensis*	Stimulation of apoptosis in breast cancer cell line	(Khorami et al. 2022)

**Fig. 5:**
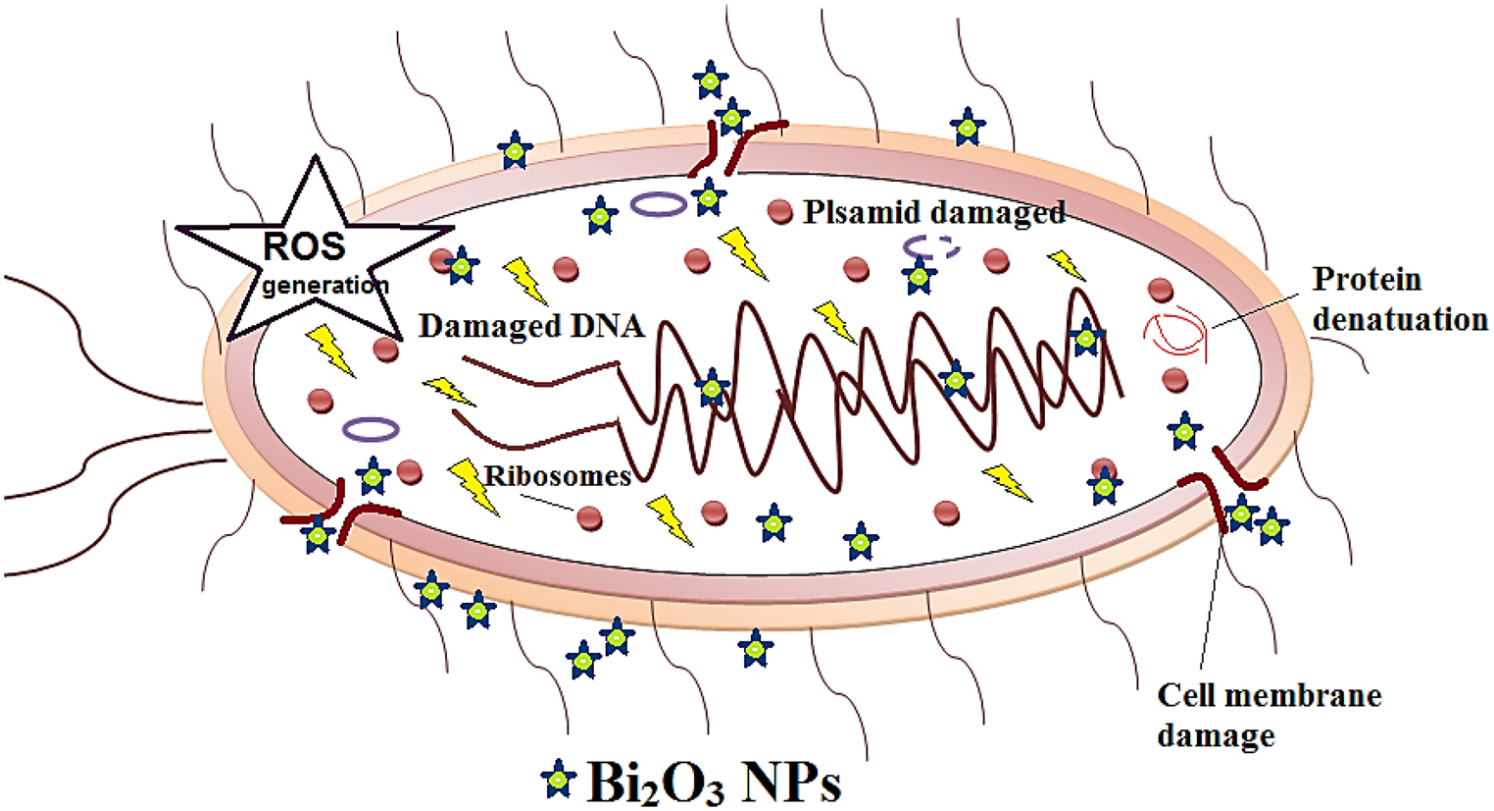
The killing mechanism of Bi_2_O_3_ NPs on bacterial cell

Conversely, it has been shown that Bi_2_O_3_ NPs exhibit a modified type of antibacterial effect against both Gram-positive and Gram-negative bacteria (El-Batal et al. 2017). Also, (Hernandez-Delgadillo et al. 2013), stated that Bi_2_O_3_ NPs displayed antifungal and antibacterial action in lowering concentrations. Bismuth oxide nanoparticles (NPs) are needed for treating drug-resistant bacteria because they prevent the growth of harmful bacteria by producing oxygen radicals that affect the Krebs cycle and the metabolism of nucleotides and amino acids. (Hosny et al. 2024) clarified that managing infectious diseases has become increasingly difficult due to the rise in multidrug-resistant organisms. To effectively treat antibiotic-resistant bacteria, novel antimicrobial chemicals must be developed urgently. Thus, Bi_2_O_3_ NPs are motivating antagonists to combat a variety of harmful illnesses (Luo et al. 2013). Bi_2_O_3_ NPs can be used as antimicrobial agents, achieving a complete inhibition of Gram-positive (MSRA) and Gram-negative bacteria (MDR-*E. coli*) at low concentrations, ∼50 ppm (Geoffrion et al. 2021). Recent studies have demonstrated that certain Bi-based compounds and Bi NPs exhibit an antibacterial effect versus *P. aeruginosa*,* S. aureus*,* Helicobacter pylori*, and *E. coli* (Wu et al. 2023b). Furthermore, because Bi NPs interact with the bacterial cell wall, trigger intrinsic and adaptive immunological responses, produce reactive oxygen species molecules, inhibit the formation of biofilms, and promote intracellular effects, they provide a treatment alternative for MDR bacteria (AlMatar et al. 2018). (Jawad et al. 2022) found that Bi_2_O_3_ NPs unveiled more effective antibacterial activity against *Acinetobacter baumannii* and *S. aureus* over amikacin, and had a MIC that was greater than that of amikacin. The killing mechanism of Bi_2_O_3_ NPs on bacterial cell was illustrated in Fig. 5.

### The minimum inhibitory concentration (MIC) and minimum bactericidal concentration (MBC) of melanin-based Bi_2_O_3_ NPs

The MIC and MBC of melanin-based Bi_2_O_3_ NPs were determined at concentrations of 3.125 up to 100 µL as revealed in Table 3. The results indicate that the MIC of Bi_2_O_3_ NPs against *C. freundii* is 12.5 µL and MBC is 25 µL, while the MIC assessment of melanin-based Bi_2_O_3_ NPs toward *E. aerogenes* is 6.25 µg/mL and MBC is 12.5 µL. Bi_2_O_3_ NPs exhibit two distinct behaviors: they are bacteriostatic at lower concentrations and bactericidal at greater levels. In the same manner, (Geoffrion et al. 2021) estimated the MIC of Bi_2_O_3_ NPs for *E. coli* and MRSA were between 0 and 5 ppm and 5–10 ppm, respectively. The antimicrobial activity of Bi_2_O_3_ NPs against *S. aureus* and the yeast *Candida albicans* under both planktonic and biofilm formation stages was reported by (Vazquez-Munoz et al. 2020).

Bi NPs demonstrated an antibacterial impact on *Enterococcus faecalis* and *Streptococcus salivarius* with MICs of 5 and 2.5 µg/mL, respectively, according to (Rostamifar et al. 2021), showing that bismuth nanoparticles (Bi NPs) had higher antibacterial activity than chlorhexidine, with lower MIC and MBC.

**Table 3:** MIC of Bi_2_O_3_ NPs against *C. freundii* and *E. aerogenes*

Bi_2_O_3_ NPs concentrations (µL)	Zone of Inhibition (mm)	
	*C. freundii*	*E. aerogenes*
3.12	0.00^a^	0.00^a^
6.25	0.000^a^	4.03 ± 0.0577^b^
12.5	3.33 ± 0.0.305^b^	6.06 ± 0.115^c^
25	7.13 ± 0.152^c^	10.06 ± 0.115^d^
50	13.40 ± 0.360^d^	17.23 ± 0.577^e^
100	21.03 ± 0.057^e^	29.16 ± 0.115^f^
LSD	0.0	2.03

LSD, Lease significant difference

### Effect of melanin-based Bi_2_O_3_ NPs on the virulence factors of *C. freundii* and *E. aerogenes*

#### The antibiofilm activity of melanin-based Bi_2_O_3_ NPs against *C. freundii* and *E. aerogenes*

**Fig. 6:**
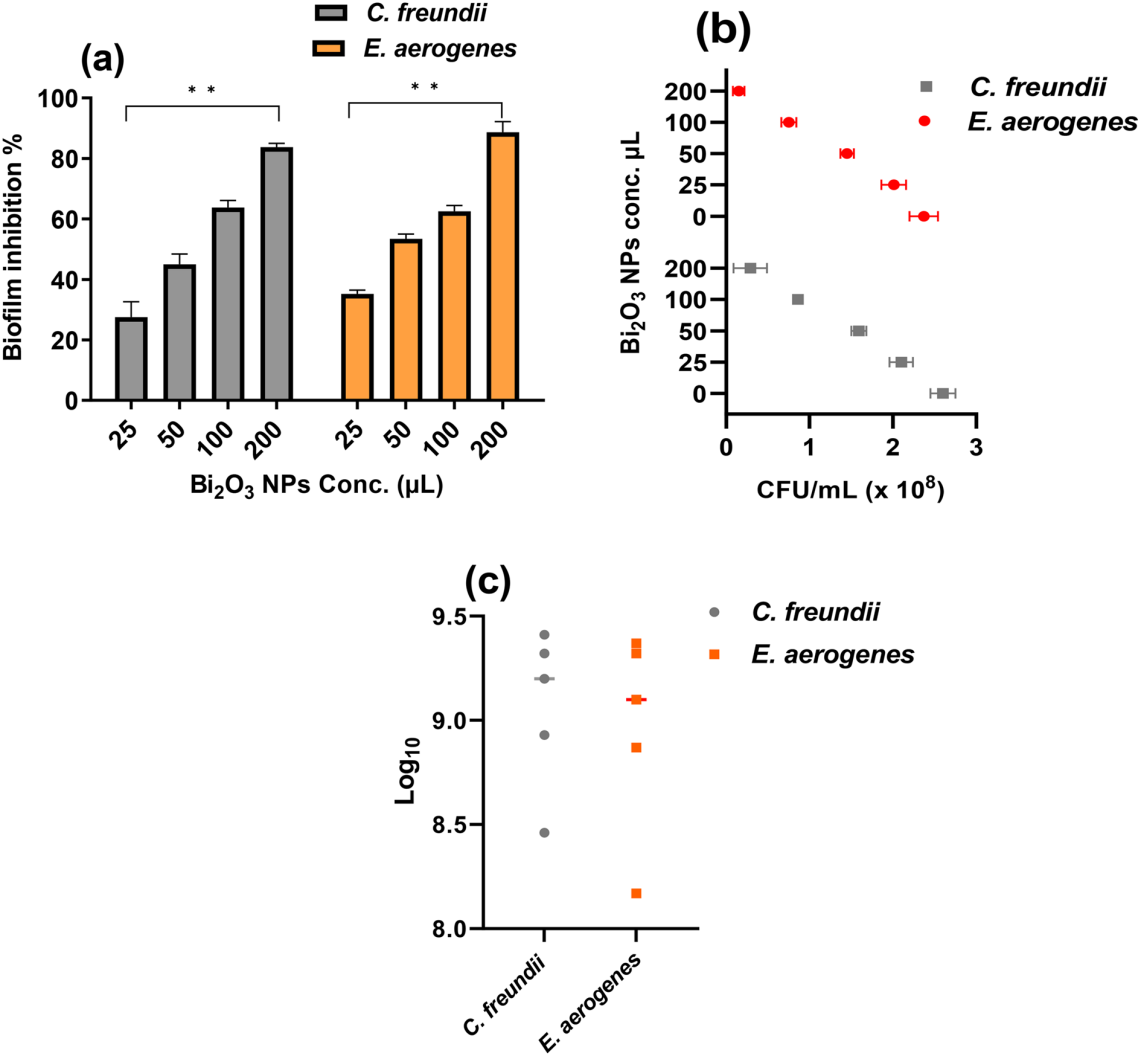
Effect of melanin-based Bi_2_O_3_ NPs at concentrations of 25, 50, 100, and 200 µL on (**a**) Biofilm formation inhibition %, (**b**) CFU/mL, and (**a**) Log_10_

Regarding the antibiofilm activity of melanin-based Bi_2_O_3_ NPs, the data presented in Fig. 6a illustrate the inhibition % of the biofilm development. The maximum suppression was obtained as 81.05 and 88.25% at a concentration of 200 µL against *C.* *freundii* and *E. aerogenes*, respectively. The biofilm is a collection of microbial cells that are encased in a matrix of mostly polysaccharides and are irrevocably attached to a material (not eliminated by ordinary rinsing) (Rashdan et al. 2023). As stated by (Janssens et al. 2008), biofilms are a predominant initiator of infection, accounting for over 80% of persistent bacterial infections worldwide. The synthesis of biofilm on the surface is explained by some mechanisms, including the existence of genes responsible for the adherence pathway and the synthesis of the exterior polymeric material (Duarte et al. 2015).

The inhibition of biofilm formation may result from that the NPs are presumably capable of reducing the transcriptional function of genes that contribute to the production of biofilm (Castro-Valenzuela et al. 2024). A few studies have shown that Bi_2_O_3_ NPs have potent antibiofilm effectiveness against a variety of pathogens in vitro. These studies have also suggested that Bi_2_O_3_ NPs have some antibacterial mechanisms, such as preventing cell division, disrupting the cytoplasmic membrane of the cell, and disturbing DNA (El-Batal et al. 2020). (Reverberi et al. 2018) reported the ability of bismuth nanoparticles to prevent the formation of *Streptococcus mutans* biofilms.

#### The impact of melanin-based Bi_2_O_3_ NPs on the viable colony counts of *C. freundii* and *E. aerogenes*

The effect of melanin-based Bi_2_O_3_ NPs at concentrations of 50, 100, 150, and 200 µL on the survival of *C. freundii* and *E. aerogenes* was assessed using a viable colony count assay. The results are presented as colony-forming units per millimeter (CFU/mL) and log_10_. The data reveal the effective repressive action of Bi_2_O_3_ NPs on *C. freundii* and *E. aerogenes* viability. A 200 µL exhibits superior antibacterial activity against *C. freundii* and *E. aerogenes* exceeding the action of other concentrations. Bacterial suppression was determined in the range of 10^–1^ to 10^–5^ ten-fold dilutions of the tested bacteria. As illustrated in Fig. 6b, CFU/mL values are strongly dependent on colony number values; therefore, CFU/mL inversely corresponds with concentrations of the treatments. Furthermore, a concentration of 200 µL gave the minimal CFU/mL (0.29 × 10^8^) for *C. freundii*, compared to (0.15 × 10^8^) for *E. aerogenes*. The CFU/mL of the untreated *C. freundii* and *E. aerogenes* are 2.60 ± 0.15 and 2.37 ± 0.17, respectively.

The findings of the colony counts are confirmed by the log_10_ values in Fig. 6c, in which 200 µL of Bi_2_O_3_ NPs yield log_10_ values of 8.46 and 8.17 for *C. freundii* and *E. aerogenes*, respectively, in contrast to the log_10_ of the untreated controls (9.41 and 9.37).

#### Effect of melanin-based Bi_2_O_3_ NPs on catalase activity (U/mL) of *C. freundii* and *E. aerogenes*

**Fig. 7:**
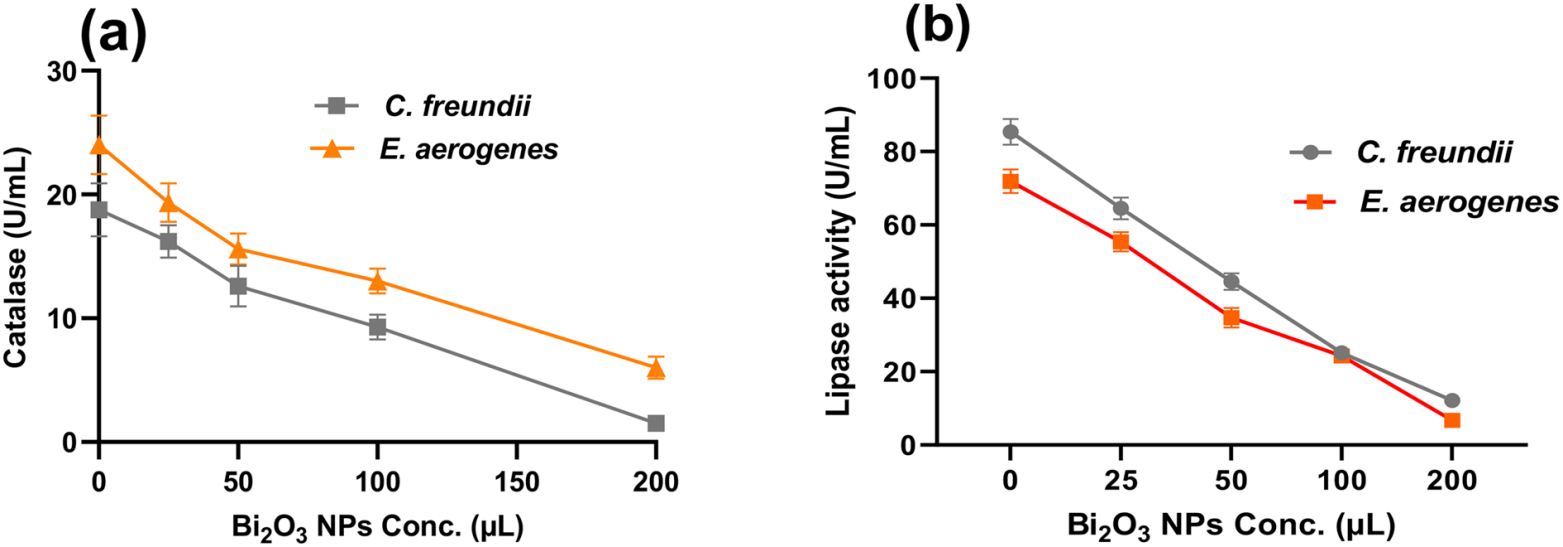
Effect of melanin-based Bi_2_O_3_ NPs at concentrations of 25, 50, 100, and 200 µL on (**a**) catalase and (**b**) lipase activities (U/mL) of *C. freundii* and *E. aerogenes*

In the current study, the inhibiting influence of melanin-based Bi_2_O_3_ NPs on catalase activity produced by *C. freundii* and *E. aerogenes* was studied. The results indicate that melanin-based Bi_2_O_3_NPs have negative impacts on the catalase activity of *C*. *freundii* and *E. aerogenes.* The inhibiting effect on catalase activity is proportional to the concentration of melanin-based Bi_2_O_3_ NPs. As shown in Figs. 7a, 200 µL of melanin-based Bi_2_O_3_ NPs greatly hinder the catalase activity, where it displays 1.5 ± 0.62 and 6.0 ± 0.91 U/mL for *C. freundii* and *E. aerogenes*, respectively, compared with the untreated control, 18.7 ± 1.73 and 24 ± 2.03 U/mL.

#### Effect of melanin-based Bi_2_O_3_ NPs on lipase activity (U/mL) of *C .freundii* and *E. aerogenes*

The inhibiting effect of melanin-based Bi_2_O_3_ NPs on lipase enzyme activity produced by *C. freundii* and *E. aerogenes* is illustrated in Fig. 7b. The results indicate that melanin-based Bi_2_O_3_ NPs have an adverse effect on the lipase activity of *C. freundii* and *E. aerogenes.* Lipase activity is inhibited in proportion to the concentration of melanin-based Bi_2_O_3_ NPs. At 200 µL of melanin-based Bi_2_O_3_ NPs, the lipase activity is inhibited to 12.13 ± 0.34 and 6.7 ± 0.26 U/mL for *C. freundii* and *E. aerogenes*, respectively, compared with the untreated controls of 85.41 ± 1.67 and 71.93 ± 1.87 U/mL.

Uropathogens produce specific virulence factors to resist the immune system of the host or invade the tissue, which are either integral components of the microbe or released by it. These factors involve microbial cell wall surface-associated components as well as secreted proteins (Soujanya and Banashankari 2023). In addition, some pathogenic bacterial species secrete lipase, which has been reported for its role as a virulence factor. *Propionibacterium acnes* and *Staphylococcus epidermidis* lipases may have a role in their invasion and survival on human skin. Moreover, several studies demonstrated that a bacterium’s potential to cause disease depends on many virulence factors and various pathogens rely on lipase and phospholipase activities (Stehr et al. 2003). These enzymes break down lipid substrates and play a variety of functions in pathogenesis, including host cell damage/modulation, signaling among cells, and inflammation (Flores-Díaz et al. 2016). Following the colonization of host cell targets, bacteria exploit lipolysis appropriately to fulfill their specific needs (Ghadimi et al. 2024).

#### Scanning electron microscopic analysis

**Fig. 8:**
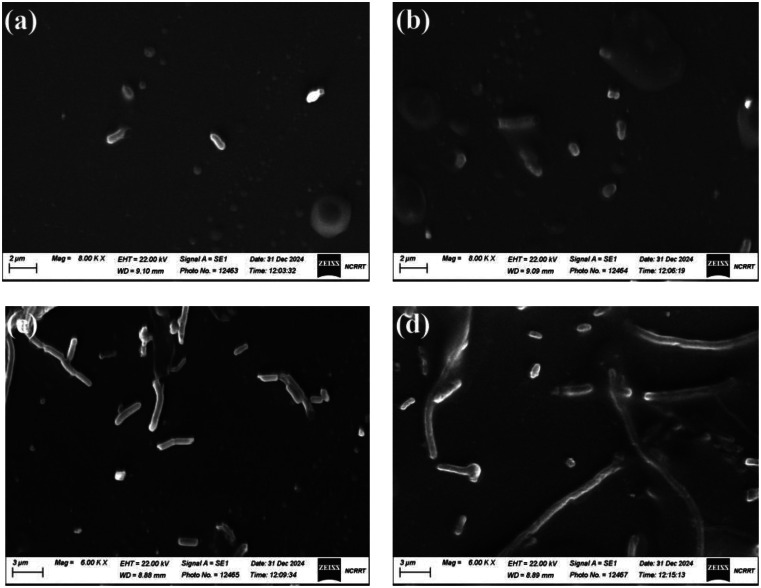
SEM images of (**a**) Untreated *C. freundii* cells, (**b**) *C. freundii* treated with melanin-based Bi_2_O_3_ NPs, (**c**) Untreated *E. aerogenes*, and (**d**) *E. aerogenes* treated with melanin-based Bi_2_O_3_ NPs

The changes in morphology observed in bacterial cells after treatment with melanin-based Bi_2_O_3_ NPs were assessed using SEM. The SEM images reveal significant morphological changes in the bacterial cells after exposure to melanin-based Bi O NPs nanoparticles. The control *C. freundii* exhibited normal rod-shaped morphology. *C. freundii* cells appear intact with a smooth surface and well-defined structure (Fig.8a). However, in the melanin-based Bi_2_O_3_ NPs treated group, contrary to normal rod-shaped cells, bacteria with irregular fragmentation developed, and the bacterial cell swelled to a larger size with substantial cell aggregation noticeable cell damage is evident, including membrane disruption, irregular shapes, and possible aggregation of nanoparticles on the bacterial surface (Fig.8b). These observations suggest that Bi O NPs nanoparticles may have induced structural alterations, potentially leading to bacterial cell death. Likewise, characteristic *E. aerogenes* had a rod, smooth appearance, and the cells were nearly identical in shape (Fig. 8c). Following exposure to melanin-based Bi_2_O_3_ NPs, the cells exhibited abrupt morphology with the disrupted outer surface (Fig. 8d). In the same manner, (Bilal et al. 2017) stated that nanoparticles can produce holes in the bacterial cell wall, disorganizing, damaging, and increasing the permeability of the cell membrane. Furthermore, NPs can affect the microenvironment around bacteria, producing ROS and causing cellular death. Electrostatic interactions between the positive charge of NPs and the negative charge of the bacterial surface resulted in a number of modifications, including membrane separation, cell distortion, and, finally, rupture of the membrane (Mousaa et al. 2022). The nanoparticles have been shown to counteract bacterial virulence factors, such as slow drug absorption, biofilm formation, and accelerated efflux. (Jawad et al. 2022) reported the antibacterial efficacy of bismuth oxide nanoparticles versus *Acinetobacter baumannii* and *Staphylococcus aureus*.

#### Cytotoxicity test of the melanin-based Bi_2_O_3_ NPs on the Vero cells using MTT protocol

**Fig. 9:**
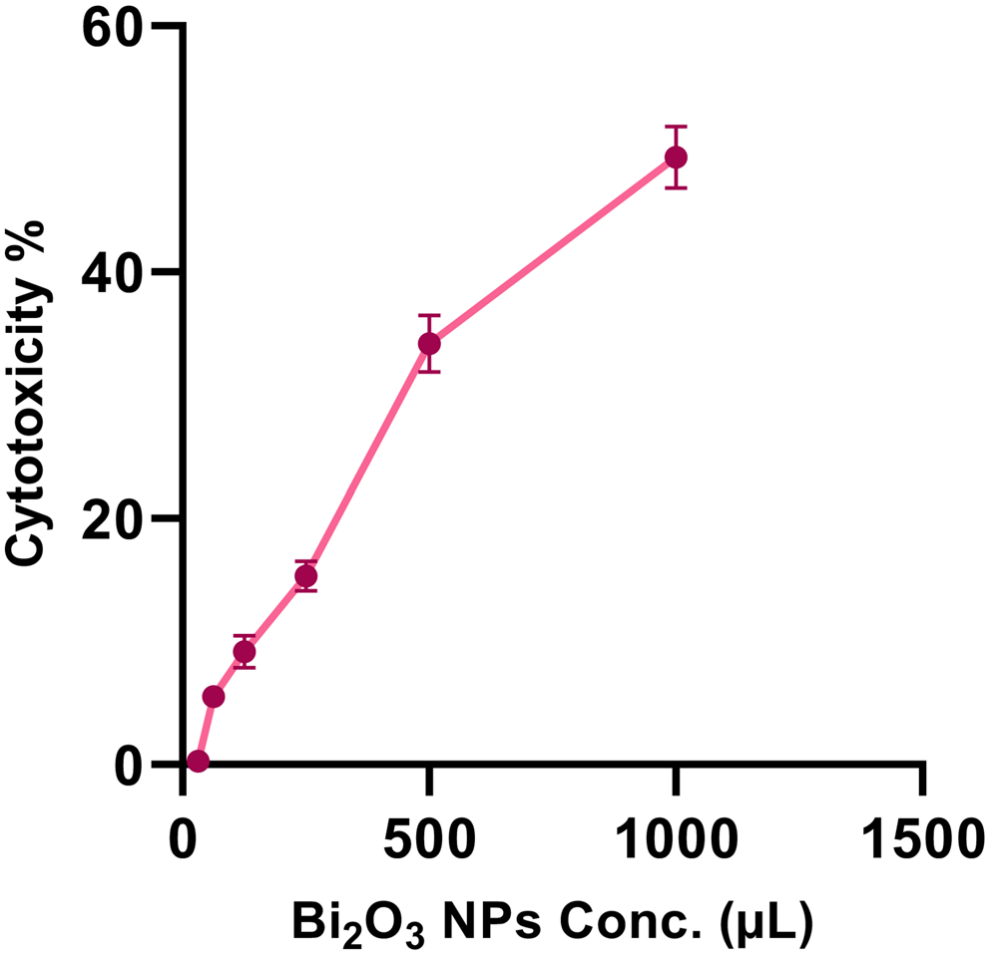
Cytotoxicity of melanin-based Bi_2_O_3_ NPs on the Vero cells

The melanin-based Bi₂O₃ NPs exhibited a dose-dependent cytotoxic effect on Vero cells, indicating moderate biocompatibility at lower concentrations. As shown in Fig. 9, the IC₅₀ value of the melanin-based Bi₂O₃ NPs against Vero cells was determined to be 513.9 ± 2.71 µg/mL, indicating a moderate cytotoxic effect on normal kidney epithelial cells. This suggests that the melanin-based Bi₂O₃ NPs maintain acceptable biocompatibility at concentrations below this threshold and may possess a therapeutic window suitable for further biomedical applications.

Similarly, (Hernandez-Delgadillo et al. 2013) demonstrated that Bi_2_O_3_ NPs had no harmful effects on monkey kidney Vero cells after 24 as compared to cells without the nanoparticles. Moreover, Cytotoxicity tests on MCF-7, KB, and HepG2 cancer cell lines showed selective activity of bismuth oxide-barium oxide (BiO-BaO) nanocomposite (IC₅₀ values between 43 and 56 µg/mL) and little toxicity to normal NHDF B cells. The improved bioactivity is due to BaO’s ability to produce reactive oxygen species (ROS) and maintain structural integrity. These findings suggest that BiO-BaO is a promising multifunctional nanomaterial for biological uses such as antibacterial treatments, drug delivery, and cancer therapy (Kaliyaperumal et al. 2025). A study conducted by (Zanganeh et al. 2025) used the MTT test to analyze the cytotoxic effects of *C. albicans*-Bi-NPs and *C. glabrata* Bi_2_O_3_-NPs on melanoma cancer cells and normal human foreskin fibroblasts (HFF). The IC_50_ values for *C. albicans*-Bi-NPs and *C. glabrata*-Bi_2_O_3_-NPs were around 184 and 173 µg/mL, respectively. These findings demonstrate that nanoparticles are more toxic to melanoma cells than to normal cells.

## Conclusion

Infectious diseases are the major cause of death worldwide and pose a risk to both the economy and public health. Additionally, they negatively impact many aspects of society and the economy. Nanomaterials hold promise as a revolutionary tool in the fight against infectious disorders. The current work highlights the green synthesis of Bi_2_O_3_ NPs utilizing fungal melanin and different doses of gamma irradiation. The maximum production was estimated at 40 kGy. Characterization of melanin-based Bi_2_O_3_ NPs indicated the production of uniform, highly stable spherical particles with a small size of 20.5 ± 1.7 nm. The antibacterial efficacy of melanin-based Bi_2_O_3_ NPs represented inhibition zone diameters of 35.26 ± 0.305 and 38.42 ± 0.570 mm for uro-pathogenic *C. freundii* and *E. aerogenes*, respectively at 200 µL. Additionally, the efficiency of melanin-based Bi_2_O_3_ NPs as an anti-virulence agent by inhibiting the formation of bacterial biofilm for *C. freundii* and *E. aerogenes* (81.05 and 88.25%, respectively). Moreover, the activities of catalase and lipase, enzymes responsible for virulence factors in uro-pathogenic *C. freundii* and *E. aerogenes*, were greatly reduced. Bacterial cell death was mostly triggered by melanin-based Bi_2_O_3_ NPs that compromised the integrity of the cell membrane. The Bi_2_O_3_ NPs can be recommended for use at low concentrations in foodstuffs, pharmaceutical applications, health care, disinfectants, and pediatric hospitals primarily for extra-intestinal pathogenic bacterial infection treatment. Despite its limitations, the study offers valuable insights into applicable methods to manage infectious diseases and recommends future research to focus on broad populations, advanced genomic approaches, and the integration of clinical data to enable effective public health tactics against antibiotic-resistant bacteria. Further perspectives are required to enhance effective approaches for Bi_2_O_3_ NPs, which require additional in vivo and clinical studies. They can also serve as drug delivery vehicles, allowing for the controlled and localized release of therapeutic medications at the infection site.

## Data Availability

No datasets were generated or analysed during the current study.

## Abbreviations

Ak: Amikacin
AMP: Ampicillin
AX: Amoxicillin
BBN: Bismuth-Based Nanoparticles
Bi: Bismuth
Bi_2_O_3_ NPs: Bismuth Oxide Nanoparticles
Bi NPs: Bismuth Nanoparticles
CAT: Catalase Activity
CFU/mL: Colony-Forming Units per Milliliter
CL: Colistin
CTR: Ceftriaxone
DLS: Dynamic Light Scattering
DO: Doxycycline
FCCFace-Centered: Cubic
FTIR: Fourier Transform Infrared Spectroscopy
GEN: Gentamicin
GN/: Gram-negative
ID-GPC: Identification positive Cocci
JCPDS: Joint Committee on Powder Diffraction Standards
KGy: Kilogray
kGyh¹: Kilogray per hour
KBr: Potassium Bromide
log_10_: Logarithm base 10
MBC: Minimum Bactericidal Concentration
MDR: Multi-Drug-Resistant
MIC: Minimum Inhibitory Concentration
Mmol: Millimole
NCRRT: National Center for Radiation Research and Technology
NPs: Nanoparticles
NV: Novobiocin
OF: Ofloxacin
PBS: Phosphate Buffer Saline
ROS: Reactive Oxygen Species
S: Streptomycin
SEM: Scanning Electron Microscopy
sp: Species
TEM: Transmission Electron Microscopy
U/mL: Units per milliliter
UTIs: Urinary Tract Infections
VA: Vancomycin
v/v: Volume/Volume
XRD: X-ray Diffraction
ZOI: Zones of Inhibition
µL: Microliter
^60^Co: Cobalt-60
